# Raman Spectroscopy as a Potential Adjunct of Thyroid Nodule Evaluation: A Systematic Review

**DOI:** 10.3390/ijms242015131

**Published:** 2023-10-13

**Authors:** Monika Kujdowicz, Dominika Januś, Anna Taczanowska-Niemczuk, Marek W. Lankosz, Dariusz Adamek

**Affiliations:** 1 Department of Pathomorphology, Faculty of Medicine, Jagiellonian University Medical College, Grzegorzecka 16, 31-531 Krakow, Poland; dariusz.adamek@uj.edu.pl; 2 Department of Pathology, University Children Hospital in Krakow, 30-663 Krakow, Poland; 3 Department of Pediatric and Adolescent Endocrinology, Institute of Pediatrics, Jagiellonian University Medical College, 31-531 Krakow, Poland; dominika.janus@uj.edu.pl; 4 Department of Pediatric and Adolescent Endocrinology, University Children Hospital in Krakow, 30-663 Krakow, Poland; 5 Department of Pediatric Surgery, Institute of Pediatrics, Jagiellonian University Medical College, 31-531 Krakow, Poland; anna.taczanowska-niemczuk@uj.edu.pl; 6 Department of Pediatric Surgery, University Children Hospital in Krakow, 30-663 Krakow, Poland; 7 Faculty of Physics and Applied Computer Science, AGH University of Krakow, Al. Mickiewicza 30, 30-059 Krakow, Poland; marek.lankosz@fis.agh.edu.pl

**Keywords:** thyroid nodule, thyroid carcinoma, thyroid dysfunction, Raman spectroscopy, diagnosis

## Abstract

The incidence of thyroid nodules (TNs) is estimated at 36.5% and 23% in females and males, respectively. A single thyroid nodule is usually detected during ultrasound assessment in patients with symptoms of thyroid dysfunction or neck mass. TNs are classified as benign tumours (non-malignant hyperplasia), benign neoplasms (e.g., adenoma, a non-invasive follicular tumour with papillary nuclear features) or malignant carcinomas (follicular cell-derived or C-cell derived). The differential diagnosis is based on fine-needle aspiration biopsies and cytological assessment (which is burdened with the bias of subjectivity). Raman spectroscopy (RS) is a laser-based, semiquantitative technique which shows for oscillations of many chemical groups in one label-free measurement. RS, through the assessment of chemical content, gives insight into tissue state which, in turn, allows for the differentiation of disease on the basis of spectral characteristics. The purpose of this study was to report if RS could be useful in the differential diagnosis of TN. The Web of Science, PubMed, and Scopus were searched from the beginning of the databases up to the end of June 2023. Two investigators independently screened key data using the terms “Raman spectroscopy” and “thyroid”. From the 4046 records found initially, we identified 19 studies addressing the differential diagnosis of TNs applying the RS technique. The lasers used included 532, 633, 785, 830, and 1064 nm lines. The thyroid RS investigations were performed at the cellular and/or tissue level, as well as in serum samples. The accuracy of papillary thyroid carcinoma detection is approx. 90%. Furthermore, medullary, and follicular thyroid carcinoma can be detected with up to 100% accuracy. These results might be biased with low numbers of cases in some research and overfitting of models as well as the reference method. The main biochemical changes one can observe in malignancies are as follows: increase of protein, amino acids (like phenylalanine, tyrosine, and tryptophan), and nucleic acid content in comparison with non-malignant TNs. Herein, we present a review of the literature on the application of RS in the differential diagnosis of TNs. This technique seems to have powerful application potential in thyroid tumour diagnosis.

## 1. Introduction

### 1.1. Clinical Aspects

The worldwide incidence of thyroid nodules (TNs; see full list of abbreviations after Conclusions) is estimated at 25%, and the risk of malignancy in TNs is 5–15% [1]. The TN’s features are assessed with ultrasonography (USG) using TIRADS scoring. Especially a single, enlarging nodule with irregular margins and psammoma bodies (small calcifications) suggests malignancy and requires further fine-needle biopsy and examination of the cytology stained with hematoxylin-eosin [2]. TNs might be caused by various changes, namely: inflammation, abscess, goiter, adenoma, changes of uncertain malignancy potential, carcinomas and, rarely, by developmental defects. Papillary (PTC), follicular (FTC) and medullary (MTC) thyroid carcinomas account for 80–90%, 5–15%, and 2% of thyroid malignancies, respectively [3]. The differential diagnosis of TN is important in planning the appropriate therapy: active surveillance or thyroid surgery. Thyroid cytology is categorized according to the Bethesda system from I to VI, where the lowest risk denotes category II (benign; 0–3%) and the highest for VI (malignant; 97–99%). In adults, the II category is recognized in 62.3% of cases, while the VI category is recognized in 7.4% [4,5,6]. The other categories have an intermediate risk of malignancy that implies under or overtreatment. TN surgery encompasses hemithyroidectomy or total thyroidectomy in non-malignant and malignant cases, respectively. Additionally, thyroid cancer requires evaluation of lymph node status and a lymphadenectomy of the central and lateral lymph node compartments. Lymph node metastases (LNM) are present in over 50% of adults and in over 80% of pediatric patients with PTC [7,8]. The thyroid gland plays an important role in human metabolism, as it produces thyroxine (T4). The most common etiology of thyroid disorders is autoimmune thyroiditis with diverse clinical manifestations: thyrotoxicosis (Graves’ disease), whereas either euthyroidism, hypothyroidism or, rarely, thyrotoxicosis can be observed in Hashimoto disease. Moreover, the occurrence of autoimmune diseases may increase after the COVID-19 pandemic [9]. In iodine-deficient countries, the insufficiency of iodine can result in goiter and hypothyroidism.

### 1.2. Raman Spectroscopy

Raman spectroscopy (RS) is a laser-based technique evoking molecular band oscillations. The energy distribution of in-elastic scattering of the light intensity from near infrared, visible, or near ultraviolet range of a known frequency and polarization is visible as a spectrum [10]. RS is a label-free, objective method used in the differential diagnosis of cellular and tissue pathologies and can be non-destructive, depending on the power and line of laser used [11,12]. The 785 nm laser line seems to work suitably even with the presence of blood, which might produce fluorescence [13,14,15]. RS is a complementary method to Fourier-Transform Infrared Spectroscopy (FTIR), which also detects oscillations of chemical groups [16]. In contrast to FTIR, RS does not require sample drying and it allows for the use of RS without any preparation of the sample, however, the sample should be secured from damage before measurement and the enhancement of the signal can be obtained for both non-specific and specific substances. RS is sensitive for lipids, amino acids, and nucleic acids and, additionally, for disulphides and cytochromes. FTIR is sensitive to protein and phosphate content and their structures, and thyroid FTIR investigation has proven to be useful in the diagnosis of TNs [17]. RS spectra are not sensitive to water content, as FTIR is, and have been used for both in vitro, for cytological and tissue samples, and in vivo analyses (fiber-optic RS performed during surgery). The RS signal can be enhanced via standing wave (plasmons) generated on nanoparticles or on the surface, and that method is named Surface-Enhanced RS (SERS) [10]. Bare nanoparticles not modified with proteins do not bind to the biological sample; therefore, SERS is considered to be label-free as the simple RS. The bands have different positions and intensities depending on the particular laser and spectrometer and also the sample type. The classification models are numerous; however, Partial Least Square Discriminant Analysis (PLS-DA), Principal Component Analysis–Linear Discriminant Analysis (PCA-LDA), the Support Vector Machine (VSM), and the Artificial Neural Network (ANN) are among the most cited [12,18,19]. The classification is based on spectral analysis in groups subjected with the reference method, and the biochemical changes are the origin of various spectral changes. The higher the number of groups in a model, the larger the database needed. Furthermore, the best classification model for a small group might be overfitting in some algorithms and not robust.

## 2. Methods

**Figure 1:**
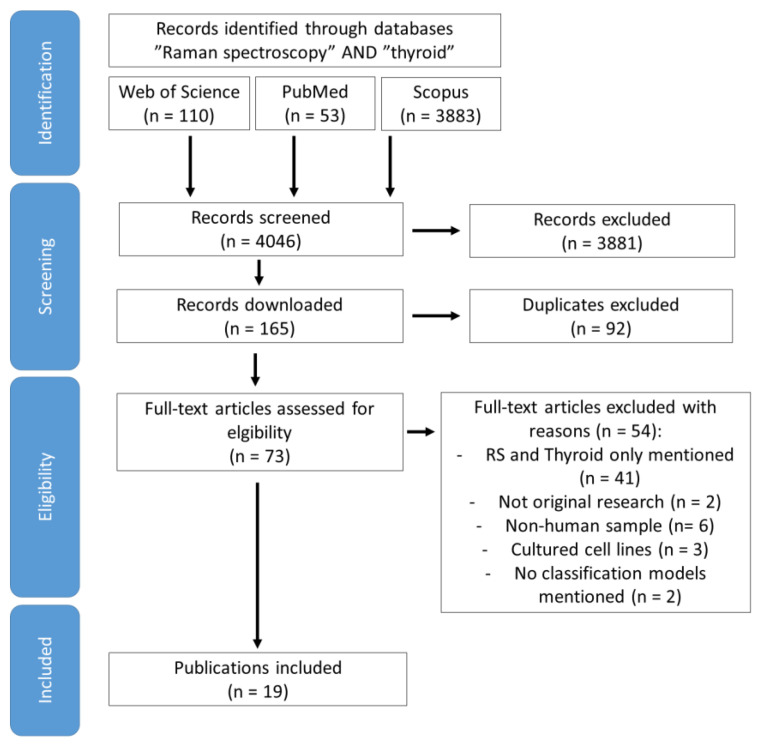
Flow diagram of studies identified for this review.

We searched the Web of Science, PubMed, and Scopus from the beginning of the databases up to the end of June 2023 using the terms “Raman spectroscopy” and “thyroid” (“thyroid” was used due to the fact not every article denoted cancer or adenoma as a thyroid nodule). Two investigators independently screened key data using a combination of key terms related to the diagnosis of TNs with RS. This systematic review has been prepared according to the PRISMA 2020 guidelines, and was not registered before the start of data extraction [20]. The inclusion criteria were original works of Raman spectroscopy (single measurements and imaging) employed for the diagnosis of TNs by building the mathematical model classifying spectra of control and thyroid tumours, both in vivo and ex vivo research on human samples with the exclusion of cell line cultures. During the screening of papers and abstracts, we decided to mention in the discussion section the interesting and useful works that did not meet the inclusion criteria but might be useful in future pharmaceutical research or for creating novel diagnostic equipment. The screened literature was downloaded to computer files, duplicates were removed, and, after that, the database was imported to Mendeley. The articles were carefully read and, due to a considerable amount of heterogeneity within, divided according to measured sample type (see flow diagram in Figure 1), listed in Table 1 and, further, reported narratively. The methodological quality of articles was biased with low patient numbers, and also different equipment and measurement parameters used in studies (it was difficult to compare results), overfitting of the models, and the reference method.

## 3. Results and Discussion

The article selection and overview shows 110 articles in Web of Science, 53 articles in PubMed, and 3883 in Scopus (see Figure 1). The different numbers of records found in different databases are related to the onset of indexing (1900, 1966, and 1971 for Web of Science, Scopus, and PubMed, respectively), and the type of data included (the broadest field is the Scopus database, which contains not only peer-reviewed academic journals and conference abstracts, but also non-peer-reviewed journals, trade magazines, books, and patents). These articles included research which mentioned “RS” or “thyroid” as an example (n = 41), non-human derived samples (n = 6), and reviews devoted to FTIR and algorithms in thyroid dyshormonogenesis (n = 2). Among all of the articles found in the databases, 24 had been performed on human samples and three of them had cultured cell lines, two did not contain the classification model, and, finally, 19 fulfilled all of the criteria of inclusion and are listed in Table 1 with division into subsections of cells, tissues (frozen and deparaffinized), and serum (dyshormonogenetic benign and malignant changes).

### 3.1. Cells from Patients

The FNA is one of the most commonly used methods to obtain thyroid cells. Thyroid fine-needle aspiration biopsy (FNAB) is performed under USG-guidance using a needle with a diameter of less than 1 mm. The obtained cytological sample is fixed, stained, and examined by the pathologist. RS can introduce an objective, semi-automatised method to detect cancer cells. We identified five studies on measurements of single thyroid human cells with RS.

Oliveira et al. (2019) reported RS imaging in the differentiation of single cells from different TNs (five benign tumours and five carcinomas) [21]. Cells derived from fresh tissue fragments were collected after collagenase digestion and subsequently fixed with formalin and measured (785 nm laser), obtaining spectra from 228 cells. The measured spectral region included 500–1750 cm^−1^ (data based on presented spectra), a pixel size of 0.5 µm, and the classification method was PCA-LDA. The main bands that were higher in PTC than in benign TN were: 1080 cm^−1^ (phosphodiester group in nucleic acids), 1260 cm^−1^ (lipids), 1293 cm^−1^ (cytosine), 1430 cm^−1^ (lipids), and 1667 cm^−1^ (amide I). In contrast, benign cells had higher bands of 1003 cm^−1^, 1031 cm^−1^, and 1362 cm^−1^ assigned to phenylamine and tryptophan (see Table 2 with the bands’ assignments). Sensitivity, specificity, and accuracy ranged from 83% to 100%. Additionally, the spectra of cells derived from single tumours representing different types of pathology (FA-20 cells, FTC-25 cells, and FV-PTC-18 cells) were collected, suggesting the feasibility of deeper discrimination.

Oliveira et al. (2020) reported measurements of cells derived from thyroid tissues, i.e., 127 benign TN, 121 PTC, and 117 MTC cells from 14 patients (four, five, and five subjects, respectively) [22]. In this study, the 785 nm laser line was used with a measured spectral region of 950–1800 cm^−1^ (data based on spectra), a pixel size of 0.5 µm, and the PCA-LDA classification method. The MTC cells exhibited an approx. two to three times higher band 1003 cm^−1^ (assigned to phenylalanine) than PTC. Furthermore, PTC had the highest 1430 cm^−1^ and the lowest 1667 cm^−1^ bands assigned to lipids and amide I, respectively. Also changes in carotenoid levels were observed. The benign and PTC versus MTC classification models showed accuracies of 97 and 99%, respectively.

Soares de Oliveira et al. (2022) classified single cells (n = 392) from 11 patients with follicular thyroid nodules [23]. In total, 392 images were taken. The classification accuracy of FA (n = 2) and Hurtle cell adenoma (HCA, currently oxyphilic cell adenoma; n = 3) versus FTC (n = 2) and Hürtle cell carcinoma (HCC, currently oxyphilic cell carcinoma; n = 11) reached 84% accuracy. In this study, the 785 nm laser line was used, the spectral region included 950–1750 cm^−1^ and a 1 µm step size, and the classification methods were PCA-LDA, SVM, and three types of ANN. Biochemical changes were not discussed.

Palermo et al. (2022) enrolled 123 patients with division into experimental groups based on USG (TIRADS scale; also inclusion criterion) and histological examination (38 benign and 85 cancerous) [24]. FNAB was performed for TIR3-TIR5, and the frozen smears placed on the glass slides were measured using a 532 nm laser. The spectral region was 100–3600 cm^−1^, the spectral resolution 1 cm^−1^ and the classification methods AHC and KM. Sensitivity and specificity were different for TIR-based grouping and RS models. The sensitivity and specificity were c.a. 60–100%. They underlined the potential clinical advantage of adding RS to FNAB to avoid unnecessary surgery.

Finally, Oliveira et al. (2019) reported RS of 77 cells extracted from 14 patients [41]. The patients were diagnosed as benign thyroid tissue, FA, FTC, and PTC, a 785 nm laser was used and the pixel size was 0.5 µm. The Principal Component Analysis (PCA; that method analyses variance when used solely) was performed and the spectra were clearly different, however, the bands were not listed and, as a result, the biochemical changes cannot be feasible.

- Oliveira et al. (2019), who reported RS of 77 cells extracted from four patients [41], can offer us additional insight into human cells spectral. The patients were diagnosed as benign thyroid tissue, FA, FTC, and PTC, a 785 nm laser was used and the pixel size was 0.5 µm. The Partial Least Square Analysis (PCA; that method analyses variance when used solely) was performed and the spectra were clearly different, however, the work did not include a classification model.

### 3.2. RS of Frozen Thyroid Tissue

The other sample type, thyroid tissue, involves cells (thyrocytes, C-cells, and others) and a tissue matrix built of collagens and other substances, as well as colloid. The fresh and frozen samples are used during thyroid surgery to plan the extension of resection (assessment of extrathyroidal extension and lymph node metastasis), and to detect the parathyroid gland with the aim to avoid postoperative hypocalcemia. RS was proven to detect healthy parathyroid and parathyroid adenoma adjacent to thyroid nerves and fat tissue [24,42,43].

Teixeira et al. (2010) reported RS measurements of 27 frozen tissue fragments from 18 patients, particularly normal thyroid tissue (11 samples), goiter nodular tissue (9), FA (1), FTC (1), and PTC (5) [25]. Spectra were collected using a 1064 nm laser. The spectral region was 450–1800 cm^−1^, the spectral resolution was 4 cm^−1^, and the classification method was Discriminant Analysis (Minitab software, Version 16, Minitab Inc., Coventry, UK). Classification accuracy was 65–73%. The spectral characteristics of the goiter were more similar to those of normal tissue. FA presented a high intensity of the 573, 690, 735, 893, and 1330 cm^−1^ bands (assigned to tryptophane and DNA). For FTC, the 678, 700, 915, 974, 980, 1224, 1290, 1485, and 1525 cm^−1^ bands (representing phosphates, amide III, and collagen) were characteristic, while the 573, 690, 735, 893, and 1330 cm^−1^ (assigned to tryptophane and DNA) were characteristic for PTC.

Rau et al. (2016) imaged frozen tissue samples from nine PTC patients (two images of tumours and two images of normal thyroids for each patient) with RS [26]. The area of the single map ranged from 0.1 × 0.1 mm^2^ to 1 × 1 mm^2^, step size 2 µm, and the 532 nm laser line was used. The spectral region was 200–3400 cm^−1^, the spectral resolution was 5 cm^−1^, the spatial resolution was 25 µm, and the classification method was PCA-LDA. The main changes were attributed to carotenoids, phenylalanine, and tryptophan (intensity of the 1006, 1156, and 1520 cm^−1^ bands). The discrimination between normal and PTC groups had a sensitivity, specificity, and accuracy of 93, 100, and 95%, respectively.

Neto et al. (2016) presented the RS of 35 frozen thyroid samples, namely: 10 normal, 10 goiter, 10 PTC, and five FTC, and showed their discrimination models based on gene expression but not RS spectra [44]. The 785 nm laser was used, the spectral region was 400–1800 cm^−1^, the spectral resolution was 4 cm^−1^, and the analysis included PCA. Additionally, the qRT-PCR gene expression of TG, TPO, PDGFB, SERPINA1, LGALS3, and TFF3 was investigated. The presence of malignancy was correlated with the larger intensities of the DNA and RNA, protein, lipids, and proline, tryptophan, and phenylalanine bands of the averaged RS spectra. Moreover, phenylalanine was higher in goiter and FTC than in healthy and PTC samples.

Medeiros Neto et al. (2017) reported an RS study (785 nm laser) on 30 frozen tissue samples, including normal thyroid (10), goiter (10), and thyroid cancer (10) [27]. The spectral region was 400–1800 cm^−1^, the spatial resolution was 2 µm, and the classification methods were PCA-LDA and Binary logistic Regression (BLR). Sensitivity and specificity were 73 and 87% for cancer versus control and 77 and 70% for goiter versus cancer, respectively. The differential vectors peaks were listed and the main differences among groups were levels of DNA, proteins (amide III and amide I) and, amino acids (proline, hydroxyproline, tryptophan, and phenylalanine), as well as lipids.

Rau et al. (2017) presented discrimination of benign tissues from follicular TN as well as FA versus FTC and the follicular variant of PTC (FV-PTC) with accuracies of 78% and 89%, respectively [28]. The 66 maps of frozen tissues from 14 patients were collected from healthy and tumoural areas with a 532 nm laser. The spectral region was 200–3400 cm^−1^ with a 25 µm confocal pinhole and a pixel size of 0.25 µm. The classification method was PCA-LDA. The authors observed higher bands 1006 and 1667 cm^−1^ in adenoma and carcinomas then in healthy tissues, and the highest 1156 and 1518 cm^−1^ bands in malignancy. The 2900 cm^−1^ band assigned to lipids was the highest in follicular adenomas.

**Figure 2:**
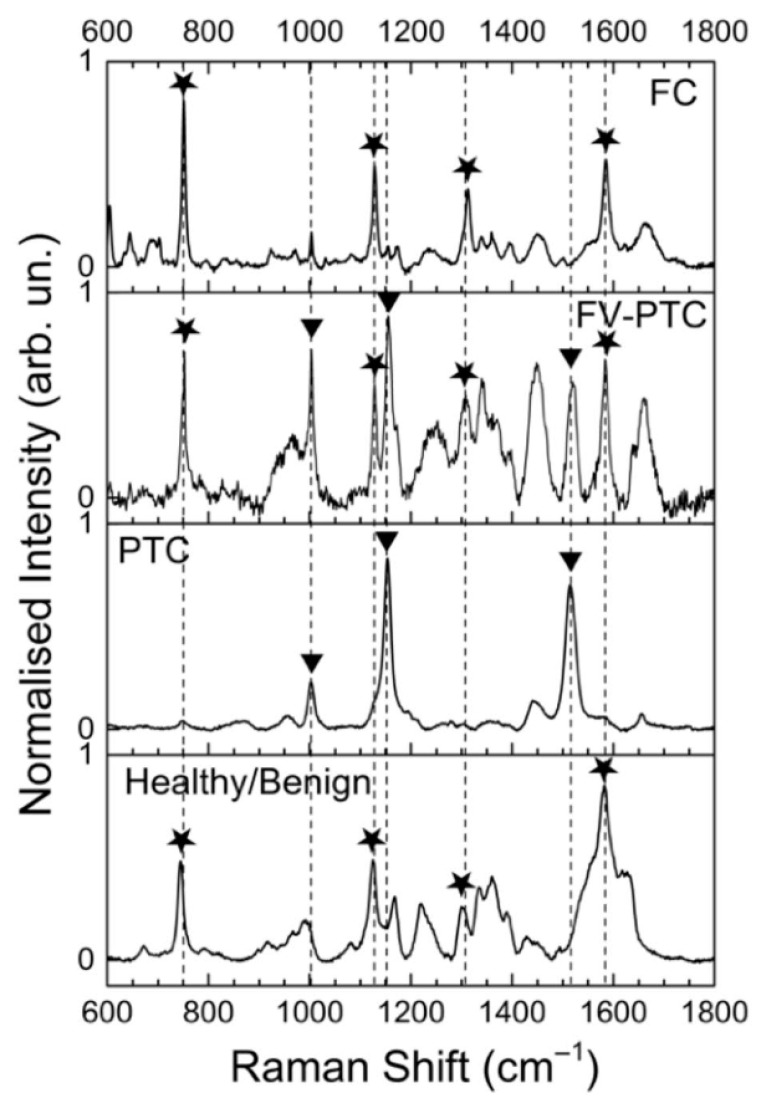
Exemplary Raman spectra (adapted from Sbroscia et al., 2020) [29]. Symbols: stars–cytochrome c, and inverted triangle–carotenoids bands.

Sbrosia et al. (2020) used postoperative frozen tissue samples from 30 patients, grouped into normal, benign, and malignant (FTC, classic PTC, and follicular variant of PTC), to obtain a classification accuracy of approx. 90% [29]. The 532 nm laser was used, the spectral region was 100–3800 cm^−1^, the spectral resolution was 1 cm^−1^, and the applied classification methods were AHC and KM. The outcomes show the pivotal role of the 1582 cm^−1^ band (present in PTC and FV-PTC). Additionally, the 747 cm^−1^ (lack in PTC), 1004 (the highest in FV-PTC), 1120 cm^−1^ (presence in FTC, FV-PTC, and benign), 1128 cm^−1^ (present in PTC and FV-PTC), and 1301 cm^−1^ (the highest in FTC) cm^−1^ bands were helpful in the differential diagnosis of thyroid pathologies (see Figure 2 with exemplary RS spectra).

Mert et al. (2022) described the SERS of frozen tissue sections [30]. In their study, colloidal silver 30 nm nanoparticles were used for RS signal enhancement. They used an 830 nm laser line and measured the spectral region 400–1800 cm^−1^. The classification method was PCA-LDA. Different “windows” were used in this study (materials on which the samples were placed) namely: glass slides, CaF_2_, and polidimethylsiloxane (PDMS). The 31 patients’ samples were divided into normal (31 samples) and tumour tissue (33 samples, benign and malignant). The classification models revealed sensitivity, specificity, and accuracy in the range of 71–100%. The most prominent changes in malignancy were DNA and RNA (724, 740, 1096 cm^−1^), lactic acid (920 cm^−1^), collagens (667 cm^−1^), proteins (1052, 1315, and 1457 cm^−1^), as well as hydroxyapatite (960, 1052 cm^−1^), possibly a component of psammoma bodies. The lipids were the lowest in benign tumours.

Wang et al. (2023) described the differentiation of FA and FTC (n = 37 in each group) based on serum and frozen tissue SERS [31]. The 785 nm laser was used. The measured spectral region included 400–1750 cm^−1^ and the classification method was Logistic Regression Analysis. The spectra from tissue revealed the impact of tryptophan and phenylalanine as well as proteins (amide III and amide I) on the differential diagnosis of the investigated groups. The serum spectra were similar in FA and FTC. Sensitivity and specificity varied between 83–100% for tissue and 50–76% for serum samples.

### 3.3. Thyroid Tissue-Deparaffinized

The deparaffinized tissue is an easily accessible source of large amounts of samples, since the paraffin-tissue blocks are archived after standard histological diagnosis. This type of sample allows for investigation of the heterogeneity and inclusion of rare types of pathologies.

Senol et al. (2018) imaged deparaffinized tissue samples from 23 patients (12 healthy and 11 with thyroid tumours) [32]. The area of one image was 10 µm^2^ and the laser line used was 785 nm, the spectral region was 100–4000 cm^−1^, and the classification method Orthogonal Partial Least Square Analysis. Sensitivity and specificity were 75% and 82%, respectively. The spectral markers were not available in this article.

Depciuch et al. (2019) measured deparaffinized tissue slides from 32 patients (FA, n = 15 and FTC, n = 17) [33]. The Attenuated Total Reflectance (ATR) FTIR and RS laser (1064 nm) were employed. The measured spectral region included 150–3700 cm^−1^ with a spectral resolution of 8 cm^−1^, and the classification method was PCA-LDA. Sensitivity and specificity were similar for both methods, and for RS they varied between 66–100%. The FTC had the lowest lipid content (2917 cm^−1^, 273 cm^−1^), and the highest level of proline (skeletal of collagen backbone, 940 cm^−1^). FA had the highest protein content (1154 cm^−1^), while healthy tissue had the highest phosphodiester level (1084 cm^−1^).

### 3.4. Thyroid Dysfunction Based on Serum RS

TNs might autonomically produce thyroxine (e.g., toxic nodules) and give symptoms of hyperthyroidism such as weight loss, tachycardia, sweating, hair loss, and trembling hands. The intensive inflammatory process, which is seen in Hashimoto disease or acute thyroiditis with abscess, might entail intensive inflammation and, in some cases, fibrosis, which, in turn, can be seen as the heterogenous echogenicity and echostructure of the thyroid parenchyma on thyroid USG. The RS can be used to investigate thyroid dysfunction [34,35,36]. Thyroid hormones contain iodine, which binds to cell membranes [45,46]. Oscillatory spectroscopies (RS and FTIR) were able to distinguish changes in the secondary structure of proteins in normal and abnormal thyroid tissues [47]. The decrease in thyroxine level in thyroid tissue caused by lithium intake was depicted in rat models [48] with the treatment of rats with TRH-altered serum RS spectra [49].

Zheng et al. (2018) described the serum RS of 34 patients with thyroid dysfunction and 40 healthy volunteers [34]. In this study, they used a 532 nm laser line and the measured spectral region was 400–1800 cm^−1^ with a spectral resolution of 0.35 cm^−1^. The classification method was PCA-SVM. The main changes were the shift of the 1513 and 1154 cm^−1^ bands. The average accuracy in different classification methods ranged from 80 to 83%.

Wang et al. (2020) reported serum samples from 95 patients with thyroid dysfunction and 90 samples from patients with normal thyroid function. The samples were measured with the use of a 532 nm laser [35]. The spectral analysis included the 400–2000 cm^−1^ region, and the classification method was several SVM algorithms. The sensitivity, specificity, and accuracy were 92, 98, and 95%, respectively. The most important differentiations were the band shifts of phenylalanine and carotenoids (C-C) groups (particularly 1003, 1155, and 1515 cm^−1^).

Tian et al. (2018) reported SERS of frozen serum from 32 patients with thyroid dysfunction and 32 with normal thyroid activity [36]. SERS spectra were collected with the use of a 633 nm laser and the diluted serum was placed on the Silmeco surface-enhanced Raman chip. The measured spectral region included 0–4000 cm^−1^ and the classification method was PCA-LDA. The highest changes were observed for the bands 1006, 1154, and 1518 cm^−1^ (C-C stretch), and also 2933 cm^−1^ (CH_2_ asymmetric stretch), which were higher in thyroid dysfunction than in euthyroid patients. Additionally, band changes of the 2900–3500 cm^−1^ region (containing O-H and N-H stretch) were listed. PCA-LDA classification models were performed and for the PC-1 vector, the model had a sensitivity and specificity of 91 and 84%, respectively.

### 3.5. Serum RS Investigations in Thyroid Carcinomas

Serum RS may contribute to the detection of thyroid cancer. Blood donation is a minimally invasive procedure, as blood is much easier to obtain than a guided biopsy. Furthermore, serum RS could possibly detect both remission and metastases.

Song et al. (2022) investigated the diagnosis of PTC based on serum RS [37]. The experimental group included 16 PTC and 32 papillary microcarcinomas (PMC). The 785 nm laser line was used, the spectral region included 123–3975 cm^−1^, and the classification methods were Ensemble Learning, the Bagging Algorithm, and the Boosting Algorithm coupled with the Decision Tree, Random Forest, and AdaBoost. The different classification models achieved an accuracy between 75–85%. The 1448 and 1668 cm^−1^ and, further, the 947 and 1003 cm^−1^ bands assigned to proteins (especially collagen and phenylalanine), were lower in PTC than in PMC

Li et al. (2014) described the SERS investigation in 77 cases (32 PTC, 20 nodular goiters, and 25 normal thyroids) [38]. The enhancement was derived from the addition of a silver nanoparticles (44 nm) solution. The tissue fragments were measured with a 785-nm laser. The measured spectral region included 400–1800 cm^−1^ with a spectral resolution of 2 cm^−1^, and the classification method was PCA-LDA. The sensitivity and specificity varied from 75 to 92%. The bands revealed multiple changes in DNA, tyrosine, tryptophane, phenylalanine, and proteins, as well as in carbohydrates and lipids levels between groups.

Liang et al. (2020) reported SERS investigations on 102 patients (70 cancers and 32 benign thyroid tumours) [39]. Colloidal silver nanoparticles (35 nm diameter) were used for enhancement. The 785 nm laser was used and the cells were filtered from the plasma pellet and frozen. The measured spectral region included 400–1800 cm^−1^ and the classification method was PLS-DA. The discrimination of benign and malignant had 84–90% accuracy (the accurate number depends on the tests used, PCA-LDA or Lasso-PLS-DA). The bands 638, 726, and 1326 cm^−1^ were higher in the benign group than in the cancer group and assigned to proteins, especially containing chemical C-S bonds and phosphates.

### 3.6. Thyroid RS Investigation In Vivo

The two works that did not include classification models are worthy to describe here briefly. We identified only one study dedicated to in vivo RS of the thyroid gland. This study presents an interesting example of the usage of RS by a surgeon intraoperatively as a promising tool for the quick assessment of thyroid pathology. Medeiros et al. (2019) investigated the in vivo intraoperative and also in vitro RS measurements with the use of a 100 µm fiberoptic (laser line 785 nm) [43]. Eight patients with PTC were enrolled in this study. The characteristic bands for cancer were 834, 947, 1255, 1451, and 1601 cm^−1^, representing proteins, carbohydrates, collagens, and phenylalanine. The spectra for in vivo and in vitro were slightly different (intensities and accurate positions), however, similar biochemical changes were observed. The PTC was characterised by the decrease in tyrosine, phenylalanine, and tryptophan and the increase in DNA and amide I. Additionally, the spectra from fat tissue and nerves were described, which is very important from a practical point of view regarding the complications of surgery. The good quality (low noise/ratio) average spectra were presented, but neither the classification models nor the spectral region and resolution are available.

### 3.7. RS on Human Cell Cultures

RS could be used as a tool for the investigation of personalized treatments, including predictive factor and dose-effects, as well as the investigation of mechanisms of drug action [50]. Three articles on RS-based research on thyroid cell lines derived from various pathologies were found during the search of database. Thyroid cell lines derived from different benign and malignant thyroid nodules had their own characteristics, and the accuracy of classification varies from 71 to 95%. The changes found in the cell lines proved to be similar to the spectral changes observed in the human thyroid. Harris et al. (2009) could discriminate two thyroid cell lines representing benign thyrocytes (Nthy-ori 3-1, 52 cells) and anaplastic thyroid carcinoma (8305C, 64 cells) with 92–95% accuracy and PCA solely with the use of a 783 nm laser line [51]. Taylor et al. (2019) presented a high-resolution (spatial resolution ~1.7 µm) study on benign cell lines (Bthy-ori 3-1) and malignant FTC (FTC-133), acquiring totally 10 images containing 49 cells [52]. They used a 532 nm laser and the measured spectral region was 200–3130 cm^−1^. The applied classification methods were the Agglomerative Hierarchical Clustering (AHC) dendrogram and K-means (KM). The accuracy of classification was 78–90% and it depended on the usage of whole-cell or middle compartment spectra. O’Dea et al. (2019) presented RS studies of thyroid neoplastic cell lines, i.e., benign (NThy-ori 3-1), PTC (K1 and TPC1), FTC (XTC1), anaplastic thyroid carcinoma (8505C and C643), and MTC (CRL-1803TT and MZCRC1) [40]. Cells were air-dried and measured with a 532 nm laser with a spatial resolution of 100 µm, and the PCA-LDA classification method was used. Sensitivity, specificity, and accuracy varied between 71–93%. Spectral markers differentiating benign and thyroid cancer cell lines were bands that represented differences in the biochemical composition of carbohydrates, nucleic acids, lipids, and proteins.

### 3.8. RS Perspectives

RS is a powerful technique broadly investigated in science, and also currently used in product quality analysis in the pharmaceutical, materials, and food industries, and in clinics for the recognition of infection factors [12,53,54,55]. The costs depend on sample preparation (e.g., the usage of antibodies conjugated with markers, and dedicated platforms that might contain silver nanoparticles), equipment, the time needed to measure by trained personnel, and programs for analysis. The RS can be used for many types of sample types and malignancies but still, there is a gap for tumours of uncertain malignant potential and for pediatric patients in which the genetical background is more complex and the metastases more often than in adults. From our experience, in bladder and breast cancer, the lymphocytic inflammation, different tissue matrix response (f.e. fibrosis), and very small content of malignant cells might be the source of misdiagnosis, but sometimes it indicates a different prognosis [56,57,58,59,60,61]. There is no investigation into tumour nodule differences in patients with and without autoimmune disorders. The advantage of the thyroid is that it has quite a homogenous structure of all the organs in comparison to the layered structure of the bladder (epithelium, fibrous subepithelial tissue, and muscle layer) and other organs, since the structures might have different spectral characteristics. The difficulty in the thyroid is hormonal activity and blood supply. The benign changes that might occur can be elusive; Teixeira et al. (2010) have shown the hardship in distinguishing healthy from goiter, and even goiter and FA were more easily distinguished from follicular-derived carcinomas (FTC and PTC) than healthy and goiter versus PTC, which indicates a high variance in healthy frozen tissue [25]. The goiter diagnosis might be easier using serum measurements with SERS, which was performed by Li et al. (2014) [38]. The serum RS has a promising accuracy (85–90%) in the classification of a normal thyroid, goiter, benign TN, PTC, and PMC. However, the experiment performed by Wang et al. (2023) revealed that the tissue SERS method appeared to be better than tissue standard RS and serum SERS for thyroid tumours, showing a better classification power reaching 100% [31]. Investigations in deparaffinized tissue and fresh frozen samples (standard RS) showed similar sensitivity and specificity (c.a. 73–94%) for the classification of malignant tumours vs. a healthy control. The usage of radiological assessment, USG-based TIRADS scale, as a reference method for RS investigation could lead to failure (low sensitivity and prevalence), and it is important to perform grouping based on histological examination [20]. The histology-based grouping models’ accuracy of classification in RS cells spectra is c.a. 99% for MTC, and 84–100% for other changes. The TI-RADS-based RS models’ sensitivity and specificity are approx. 60–100%. Frozen tissue classification models of normal, goiter, benign TN, FA, FTC, PTC, and FV-PTC showed an accuracy of up to 100%. The rare subtypes of thyroid cancer have still not been investigated (i.e., solid/trabecular or tall-cell variants of PTC) however, Rau et al. (2016) proved worse classification parameters for classic versus follicular PTC variants than healthy from PTC [26], but in—Oliveira et al. (2019), the classification parameters for variants (CV-PTC vs. FV-PTC) were slightly better than the classification of CV-PTC from benign [21]. Rau et al. (2017) showned the discrimination of FA from both FTC and PTC [28] and Depciuch et al. (2019) showed the discrimination of FA from FTC [33]. We did not find a report classifying PTC from FTC nor anaplastic carcinoma, possibly due to the low incidence rate of non-PTC cancers which have worse outcomes. Soares de Oliveira et al. (2020) showed the better discrimination from benign of medullary carcinoma than papillary carcinoma, and it is possibly related that MTC stems from cells with neural differentiation (which produces calcitonin) rather than PTC, which is a follicular-derived cancer (cells stem from thyrocytes, which produces colloid with thyroxine) [21]. The oxyphilic cells change cases (Hürtle cell adenoma and Hürtle cell carcinoma) were included in - Oliveira et al. (2022) and Rau et al. (2017) and, among them, Oliveira et al. (2022) reported classification of both the oxyphilic tumours from FA and FTC (all four groups in a model of SVM and a model of CNN with similar accuracy of both classification algorithms) [27,28]. The Soares de Oliveira et al. (2022), Zheng et al. (2018), Wang et al. (2020), Song et al. (2022), and Liang et al. (2022) studies tried to employ different analysis types (SVM, PLS, and LDA subtypes) for the obtained RS spectra [27,34,35,37,39]. The fully acknowledged evaluation of mathematic algorithms of spectra classification could be assessed only in a large-scale study, and there still is a lack of such a type of investigation. It is also important to develop algorithms according to the application of a tool highly sensitive for screening (cells and serum samples; false positives are bearable here in spite of false negatives), and highly specific for the accurate diagnosis of tissue samples.

## 4. Conclusions

The PubMed, Web of Science, and Scopus search identified studies presenting promising outcomes for the implementation of RS in the diagnosis of benign and malignant TNs. Thyroid dysfunction can be detected with serum RS with 80–95% accuracy. Cells derived from tissues and measured in smears can be included in samples from benign TNs, FA, HCA, FTC, PTC, HCC, and MTC, however, classification models were not presented for all cancer subtypes. The classification parameters of patients’ cells RS and fresh frozen tissue SERS spectra are 81–100%, for models with grouping based on histology. The RS technique can be used for thyroid dysfunction detection (serum samples), screening tests (cells from patients), and the precise diagnosis of tumours (tissue samples, especially SERS). The most important chemical changes include an increase of amino acids, proteins (different bands and their structure), collagens, and nucleic acids in malignancy. Carbohydrate, carotenoid, and lipid changes play a minor role. The usage of different laser lines, detectors, and spectrometers and, further, the detection of different biochemical changes leads to similar classification parameters. It seems that a 785 nm laser is the best choice for thyroid disease, both due to a high number of reports, and the low fluorescence of blood when using this laser line. The accuracy did not vary as much when the spectral resolution and spectral region were different. The most important element in good classification is the gold standard grouping method, sample type, and appropriate chemometric analysis, including preprocessing, data reduction, and a robust classification method. Future studies on large groups of patients using multiple RS methods are needed to optimize the diagnosis of TNs, and also to specify prognosis. Furthermore, in vitro investigation could yield a predictive value for personalized treatment.

## Figures and Tables

**Table 1:** The subject and classification parameters of patient samples. All models included fingerprint region. We are not able to fully assess the quality of spectra (different equipment (lasers and spectrometers, different spectral resolution (not complete data) and, moreover, the low number of experimental groups).

Subject	Laser Line and Power	Spectral Region and Resolution [cm^−1^]	Classification Model	Classification Parameters	Author, Year, Ref.
Cells From Patients					
10 patients: 5 PTC and 5 benign TNs (divided into subtypes); 121 and 127 cells of PTC and benign, respectively (228 cells in total)	785 nm; 2 W	500–1750; n.a.	PCA-LDA	Se 95%, Sp 97%, Acc 97% (CV-PTC vs. benign) Se 100%, Sp 100%, Acc 100% (FTC vs. FA) Se 94%, Sp 98%, Acc 98% (CV-PTC vs. FV-PTC)	- Oliveira et al., 2019 [21]
14 patients: 4 benign, 5 classic variant PTC, 5 MTC; 365 cells in total	785 nm; n.a.	950–1800; n.a.	PCA-LDA and 1003 cm^−1^ band intensity	PLS-DA: Se 96%, Sp 95%, Acc 95% (MTC vs. benign) Se, Sp–n.a, Acc 79% (PTC vs. benign) Se 99%, Sp 99%, Acc 99% (MTC vs. PTC) Based on 1003 cm^−1^ intensity: Acc 87% (MTC vs. benign) Acc 79% (PTC vs. benign) Acc 95% (MTC vs. PTC)	- Oliveira et al., 2020 [22]
11 patients: 2 FA, 3 HCA, 2 FTC, 4 HCC; 392 cells in total	785 nm; n.a.	950–1750; n.a.	PCA-LDA, SVM, ANN	Acc for PCA-LDA models c.a. 60% Acc c.a. 81% (SVM, CNN, all 4 groups in one model) Acc 84% (SVM, malignant vs. non-malignant) Acc 83% (CNN malignant vs. non-malignant)	- Oliveira et al., 2022 [23]
123 patients: 38 benign, 85 cancer (patients from TIR3A to TIR5 in USG)	532 nm; 100 mW	100–3600; 1	AHC, KM	Se 60–85%, Sp 87–100%, Prevalence 27–80%	Palermo et al., 2022 [24]
Frozen Tissue					
18 patients, 27 tissue samples: healthy (11), goiter (9), FA (1), FTC (1), PTC (5)	1064 nm; 300 mW	450–1800; 4	Discriminant Analysis of Minitab software	Acc 58% (goiter TNs vs. healthy) Acc 65% (healthy and goiter vs. PTC) Acc 73% (goiter and FA vs. PTC and FTC)	Teixeira et al., 2010 [25]
9 PTC patients: 7 CV-PTC, 3 FV-PTC; 2 healthy and 2 cancer images of each patient (1 patient had mixed variant CV/FV-PTC and additional 2 maps)	532 nm; 8 mW	200–3400; 5	PCA-LDA	Se 100%, Sp 100%, Acc 100% (healthy vs. PTC) Se 93%, Sp 100%, Acc 95% (CV-PTC vs. FV-PTC)	Rau et al., 2016 [26]
30 samples (number of patients n.a.): 10 normal thyroid, 10 goiter, and 10 thyroid cancer (7 PTC and 3 FTC)	785 nm; 20 mW	400–1800; n.a.	PCA-LDA with cross validation and binary logistic regression (BLR) analysis	Se 73%, Sp 87 %, Acc 89% (cancer vs. control); Se 77%, Sp 70%, Acc 86% (goiter vs. cancer) Se 60%, Sp 67%, Acc 81% (normal vs. goiter)	Medeiros-Neto et al., 2017 [27]
14 patients: 5 FV-PTC, hyperplastic 1 TN, 4 FA, 2 HCA; 66 maps in total from tumours and healthy tissue	532 nm; 8 mW	200–3400; 5	PCA-LDA	Se 89%, Sp 68%, Acc 78% (healthy vs. follicular neoplasms); Se 93%, Sp 86%, Acc 89% (FA vs. FTC and PTC)	Rau et al., 2017 [28]
30 patients, 46 samples: 15 healthy, 8 FA, 3 FTC, 15 PTC, 4 FV-PTC	532 nm; 60 mW	100–3800; 1	AHC, KM	Acc 90% (healthy vs. cancers) Acc 82% (healthy vs. all tumours)	Sbroscia et al., 2020 [29]
SERS of 31 patients, 64 samples: 31 healthy and 33 tumours (22 benign and 11 malignant)	830 nm; 30 mW	400–1800; n.a.	PCA-LDA	Se 94%, Sp 79%, Acc 93%, (benign and malignant) Se 79%, Sp 71%, Acc 76% (benign and healthy) Se 100%, Sp 76%, Acc 91% (malignant and healthy)	Mert et al., 2022 [30]
SERS of 74 patients: 37 FA, 37 FTC	785 nm; n.a.	400–1750; n.a.	Logistic regression analysis	Se 100%, Sp 83–100%, Acc 100% (tissue) Se 75–88%, Sp 50–57%, Acc 61–68% (serum)	Wang et al., 2023 [31]
Deparaffinized Tissue					
23 samples (number of patients n.a.): healthy (12), tumours (11)	785 nm; n.a.	100–4000; n.a.	OPLS	Se 75%, Sp 82%	Senol et al., 2018 [32]
32 patients: 15 FA, 17 FTC	1064 nm; 1 W	150–3700; 8	PCA-LDA	Se 76%, Sp 81% (control vs. FTC) Se 81%, Sp 94% (control vs. FA) Se 66%, Sp 70% (FA vs. FTC)	Depciuch et al., 2019 [33]
Serum–TNs with Thyroid Disorders					
74 patients: 34 with thyroid dysfunction and 40 healthy	532 nm; 100 mW	400–1800; 0.35	PCA-SVM (AFSA, AFUD, GS)	Acc: AFUD-SVM 83%, GS-SVM 81%, AFSA-SVM 80%	Zheng et al., 2018 [34]
185 patients: 95 with thyroid dysfunction and 90 patients with normal thyroid function	532 nm; 50 mW	400–2000; n.a.	SVM (PSO, SAPSO, GA, AFUD, GS, PLS-GAPSO)	The best GAPSO-SVM: Se 92%, Sp 98%, Acc 95%	Wang et al., 2020 [35]
SERS of 64 patients: 32 with thyroid dysfunction and 32 with normal thyroid function	633 nm; n.a.	0–4000, n.a.	PCA-LDA	Se 91%, Sp 84%, Acc 87%	Tian et al., 2018 [36]
Serum-cancer					
48 patients: 16 PTC, 32 PMC	785 nm; 280 mW	123–3975; n.a.	DT, RF, and Adaboost	Acc: DT 75%, RF 82%, Adaboost 85%	Song et al., 2022 [37]
SERS of 77 patients: 32 PTC, 20 nodular goiter, 25 healthy	785 nm; n.a.	400–1800; 2	PCA-LDA	Se 92%, Sp 83% (normal) Se 75%, Sp 89% (nodular goiter) Se 88%, Sp 84% (PTC)	Li et al. 2014 [38]
SERS of 102 patients: 70 cancers and 32 benign thyroid tumours	785 nm; 2 mW	400–1800; n.a.	PCA-LDA, Lasso-PLS-DA	PCA-LDA: Se 61–84%, Sp 44–53%, Acc 84% Lasso-PLS-DA: Se 90%, Sp 59%, Acc: 80%	Liang et al. (2020) [39]

Abbreviations: TNs—thyroid nodules; FA—follicular adenoma; HCA—Hürtle cell adenoma (oxyphilic adenoma); PTC—papillary thyroid carcinoma; PMC—papillary microcarcinoma; FTC—follicular thyroid carcinoma; MTC—medullary thyroid carcinoma; HCC—Hürtle cell carcinoma (oxyphilic carcinoma); FV-PTC—follicular variant of PTC; n.a.—not applicable; Se—sensitivity; Sp—specificity; Acc—accuracy; vs.—versus; SERS—surface-enhanced RS; PLS-DA—Partial Least Square Discriminant Analysis; OPLS—Orthogonal PLS; PCA-LDA—Principal Component Analysis–Linear Discriminant Analysis; SVM—Support Vector Machine; ANN—Artificial Neural Network; KM—K-means; AHC—Agglomerative Hierarchical Clustering; DT—Decision Tree; RF—Random Forest; PSO—particle swarm optimization; GAPSO—genetic PSO; GS—grid search-based; GA—genetic algorithm; AFSA—artificial fish swarm algorithm; AFUD—artificial fish coupled uniform design; and SAPSO—simulated annealing particle swarm optimization.

**Table 2:** Assignments of the main RS bands in thyroid studies (400–1800 cm^−1^ region) [25,26,27,29,40].

Band [cm^−1^]	Assignment
430	Cholesterol ester
454	Ring torsion of phenyl
477	Polysaccharides
509–524	S-S disulfide stretching
573	Tryptophan, cytosine, guanine
600	Nucleotides
673	Tryptophan (ring breathing)
700	Methionine
720	DNA and RNA
747	Cytochrome c
816	Collagen, ν(C-C)
856	Collagen protein, proline hydroxyproline, tyrosine
918	Proline, hydroxyproline, lactic acid
940	Proline, hydroxyproline, collagen ν(C-C)
957	Carotenoids, phosphates, cholesterol
1004–1006	Phenylalanine, νs(C-C), carotenoids
1032	τ(HCH)(CH_3_), τ(HCH)(CH_2_) collagen, phospholipids, phenylalanine
1084	Phosphodiester groups in nucleic acids
1138	ν(C-N) of proteins and ν(C-O) of carbohydrates
1156	ν(C-C), ν(C-N) of proteins and ν(C-C) of lipids, carotenoids
1172	Tyrosine, δ(C-H)
1204	Amide III, tryptophan, phenylalanine, adenine
1224	Amide III (β-sheet structure)
1242–1266	Amide III of collagen, CH_2_ wagging, ν(C-N), proteins (α-helix), pyrimidine bases
1282	Collagen, nucleic acids, phosphates
1304	τ(HCH)(CH_3_), τ(HCH)(CH_2_) collagen
1312	CH_2_, CH_3_ twisting mode of collagen/lipid
1360	Tryptophan
1370	Carbohydrates
1440	δ(CH_2_, CH_3_), lipids, cholesterol
1480	Guanine, adenine (ring breathing of DNA bases)
1526	In plane vibrations of conjugated (C=C) carotenoids
1545–1568	Tryptophan
1592–1602	δ(C=C), phenylalanine
1636	Amide I, collagen
1660	Amide I (α-helix), ν(C-C)cis fatty acids
1664	Amide I, collagen, lipids, DNA
1684	Amide I ν(C=O)

Vibration modes: ν–stretching; δ–bending; τ–twisting; and s–symmetric.

## Abbreviations

TNs—thyroid nodules; FA—follicular adenoma; HCA—Hürtle cell adenoma (oxyphilic adenoma); PTC—papillary thyroid carcinoma; PMC—papillary microcarcinoma; FTC—follicular thyroid carcinoma; MTC—medullary thyroid carcinoma; HCC—Hürtle cell carcinoma (oxyphilic carcinoma); FV-PTC follicular variant of PTC, FNAB—Fine-Needle Aspiration Biopsy; USG—Ultrasonography; RS—Raman spectroscopy; SERS—surface-enhanced RS; n.a.—not applicable; Se—sensitivity; Sp—specificity; Acc—accuracy; vs.—versus; PLS-DA—Partial Least Square Discriminant Analysis; OPLS—Orthogonal PLS; PCA-LDA—Principal Component Analysis–Linear Discriminant Analysis; SVM—Support Vector Machine; ANN—Artificial Neural Network; KM—K-means; AHC—Agglomerative Hierarchical Clustering; DT—Decision Tree; RF—Random Forest; PSO—particle swarm optimization; GAPSO—genetic PSO; GS—grid search-based; GA—genetic algorithm; AFSA—artificial fish swarm algorithm; AFUD—artificial fish coupled uniform design; and SAPSO—simulated annealing particle swarm optimization.
